# Spiny Mice Modulate Eumelanin to Pheomelanin Ratio to Achieve Cryptic Coloration in “Evolution Canyon,” Israel

**DOI:** 10.1371/journal.pone.0008708

**Published:** 2010-01-14

**Authors:** Natarajan Singaravelan, Tomas Pavlicek, Alex Beharav, Kazumasa Wakamatsu, Shosuke Ito, Eviatar Nevo

**Affiliations:** 1 Institute of Evolution, University of Haifa, Mount Carmel, Haifa, Israel; 2 Department of Chemistry, Fujita Health University School of Health Sciences, Toyoake Aichi, Japan; Lund University, Sweden

## Abstract

**Background:**

Coat coloration in mammals is an explicit adaptation through natural selection. Camouflaging with the environment is the foremost evolutionary drive in explaining overall coloration. Decades of enquiries on this topic have been limited to repetitive coat color measurements to correlate the morphs with background/habitat blending. This led to an overwhelming endorsement of concealing coloration as a local phenotypic adaptation in animals, primarily rodents to evade predators. However, most such studies overlooked how rodents actually achieve such cryptic coloration. Cryptic coloration could be attained only through optimization between the yellow- to brown-colored “pheomelanin” and gray to black-colored “eumelanin” in the hairs. However, no study has explored this conjecture yet. “Evolution Canyon” (EC) in Israel is a natural microscale laboratory where the relationship between organism and environment can be explored. EC is comprised of an “African” slope (AS), which exhibits a yellow-brownish background habitat, and a “European” slope (ES), exhibiting a dark grayish habitat; both slopes harbor spiny mice (*Acomys cahirinus*). Here, we examine how hair melanin content of spiny mice living in the opposing slopes of EC evolves toward blending with their respective background habitat.

**Methodology/Principal Findings:**

We measured hair-melanin (both eumelanin and pheomelanin) contents of 30 spiny mice from the EC using high-performance liquid chromatography (HPLC) that detects specific degradation products of eumelanin and pheomelanin. The melanin pattern of *A. cahirinus* approximates the background color of the slope on which they dwell. Pheomelanin is slightly (insignificantly) higher in individuals found on the AS to match the brownish background, whereas individuals of the ES had significantly greater eumelanin content to mimic the dark grayish background. This is further substantiated by a significantly higher eumelanin and pheomelanin ratio on the ES than on the AS.

**Conclusion/Significance:**

It appears that rodents adaptively modulate eumelanin and pheomelanin contents to achieve cryptic coloration in contrasting habitats even at a microscale.

## Introduction

The study of phenotypic responses to the environment embraces a long, enlightened history [1]–[3]. A pattern of geographic variations in the phenotype provides evidence for natural selection [4], [5]. Such geographic variations invoke spatially differing selection and thus provoke varying levels of local adaptations. A classic example of local adaptation of the phenotype is concealing coloration in animals [5]–[9]. Melanism is one form of concealing coloration, prevailing in a wide array of organisms [10]. Coat/pelage color variation in small mammals is a credible and convincing example of phenotypic variation in response to natural selection in different environments; many species closely match the color of the substrate on which they live. This geographic variation in a phenotype is well documented both within and between species. Examples of intraspecific color variation in rodents include the canyon mouse (*Peromyscus crinitus*) [11], the deer mouse (*Peromyscus maniculatus;*) [12], the oldfield mouse (*Peromyscus polionotus*) [13], and the rock pocket mouse (*Chaetodipus intermedius*) [7].

Animals attain concealment when overall body color resembles their background in which they live [8], [14]. Such background matching is technically known as crypsis [15]. Coloration in mammals *per se* has been hypothesized to serve three main adaptive functions: intraspecific communication, concealment (crypsis), and regulation of physiological processes such as thermoregulation, UV protection, etc. [16]. Geographical variation in coloration is largely thought to be attributable to crypsis and thermoregulation. In addition to striking variations like melanic and non-melanic morphs, there are local color races among mammals with a gradational series of pale, intermediate, and dark colored-races on pale, intermediate, and dark-colored background types [8]. It is not uncommon to notice certain color forms occurring in close proximity but under almost identical climatic conditions that closely harmonize with two extreme types of background, e.g., black lava and white sand [17]. Altogether, color variation is evident both at macro- and microgeographical levels. While climatic clines and crypsis appeared as factors influencing macrogeographic color variations, local and non-climatic color-adaptation is primarily for crypsis.

There is ample evidence documenting what proportion of functional colors and patterns represent background color-matching [18]–[24]. There have been cross-species and cross-generic studies restricted to a repeated claim that cryptic coloration adaptation exists, ignoring how the adaptation develops. The molecular basis by which the rodents attain the match of their geographical background has been revealed only recently [25]–[30]. However, no study, as far as we know from the literature, has elucidated the “biochemical basis” detailing how hair-melanin contents evolve in harmony with background color. Therefore, it currently is the least studied in adaptations of species to their habitats. Indeed, hair-melanin of many species of rodents is characterized by both pheomelanin, which is yellow to brown, and eumelanin, which is gray to black [31]. Therefore, theoretically, it is anticipated that the background color-matching to different colored environments could be attained through a possible modulation in pheomelanin and eumelanin contents in the hair. However, no studies have been carried out to address this hypothesis.

**Figure 1:**
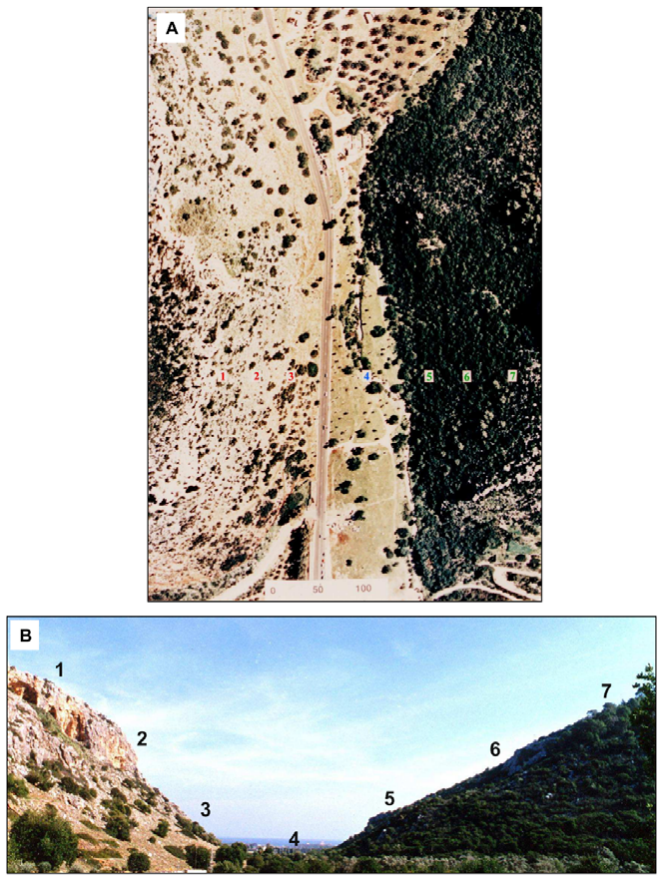
“Evolution Canyon” (EC), Nahal Oren, Israel. (A) aerial view and (B) cross-section showing the opposing slopes. Note the two habitat types; dry, open habitat (with light colored background) on the “African” slope and humid, closed habitat (with dark-colored background) in the “European” slope.

The “Evolution Canyon” model [32]–[35] is a natural microscale laboratory investigating biodiversity evolution including phenotypic and genotypic adaptation to the environment. The model has ecologically divergent environments on a microscale level (Figure 1A), imposing divergent selective pressures on the surviving organisms. The orientation and spatial structure of opposing slopes of “Evolution Canyon” (EC hereafter) cause differential insulation of solar radiation on each slope [36]. This, in turn, is driving the two opposite slopes into ecologically heterogeneous environments (Figure 1B); the tropical, open, sunny, grassy, drier south-facing slope typifies the African biota dubbed the “African” slope (AS = SFS) and the temperate, shady, lush, humid north-facing slope characterizes European biota dubbed the “European” slope (ES = NFS). The opposing slopes (separated on average by 200 m) of EC harbor spiny mice (*Acomys cahirinus*), which exhibit slope-specific adaptations to various selection pressures ([33], [37] and see also Nevo list of publications on “Evolution Canyon” linked to http://evolution.haifa.ac.il].

**Figure 2:**
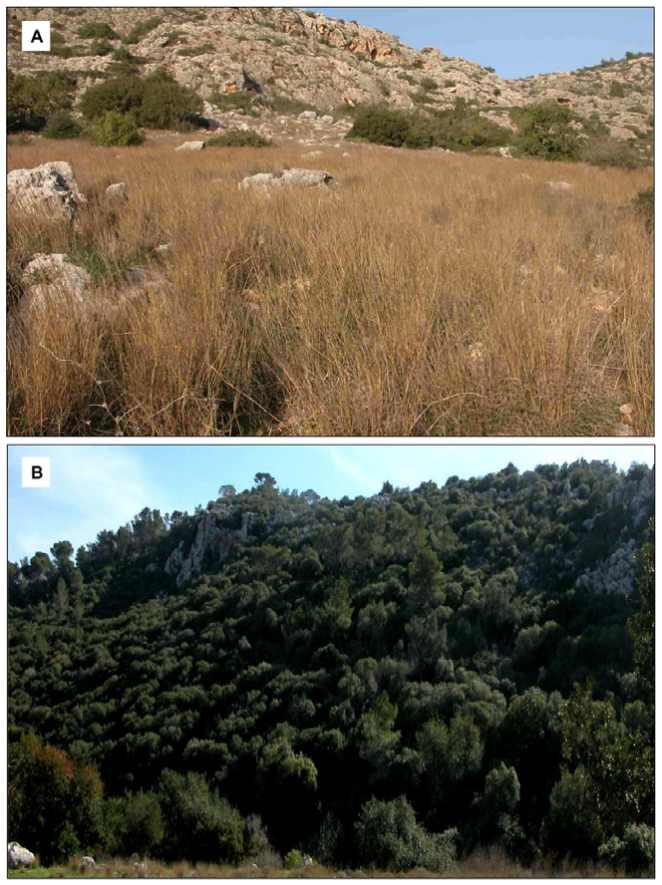
Habitat divergence in “Evolution Canyon”. (A) photo showing part of the “African” slope (AS)/south-facing slope (SFS) of EC, characterized by light terra rossa soil with a stretch of grass cover that generates a yellow-brownish background. (B) Photo of part of the “European” slope (ES)/north-facing slope (NFS), characterized by dark terra rossa soil and shady, humus-laden dark background.

**Figure 3:**
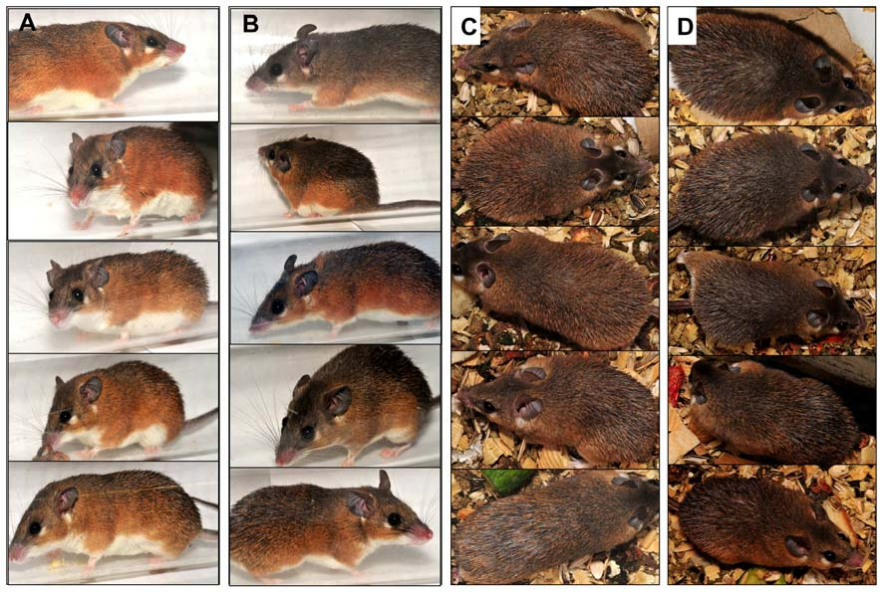
Differences in color morphs of spiny mice. (Lateral view: (A,B) and dorsal view: (C,D) inhabiting “African” slope (A,C) and “European” slope (B,D) of Evolution Canyon I, Israel.

The “African” slope is characterized by light terra rossa soil with a stretch of yellow grass cover displaying a yellow-brown colored milieu (Figure 2A); by contrast, the “European” slope is characterized by dark gray terra rossa soil with shade from the dense foliage covering trees and shrubs spread across the slope displaying a dark grayish milieu (Figure 2B) [36]. Thus, EC displays a fairly uniform color pattern of the habitats within each slope but places conflicting selection pressures on spiny mouse (*A. cahirinus*) to mimic differently colored habitats between the slopes (e.g., AS vs. ES). Individuals of *A. cahirinus* on the opposing slopes exhibit visible differences in their overall coat color: AS individuals tend to be lighter and ES individuals tend to be darker (Figure 3A–3D). Here we investigate how hair melanin contents of *A. cahirinus* evolved in response to alternative selection pressures inflicted by contrasting colored habitats of EC. Our working hypothesis is that spiny mice would exert a relatively higher pheomelanin content on the AS (than on the ES) where the demand is to mimic yellow-brown backgrounds and higher eumelanin content on the ES (than on the AS) where the demand is to mimic dark gray backgrounds to increase survival fitness by reducing the potential to be detected by predators.

### Results

**Figure 4:**
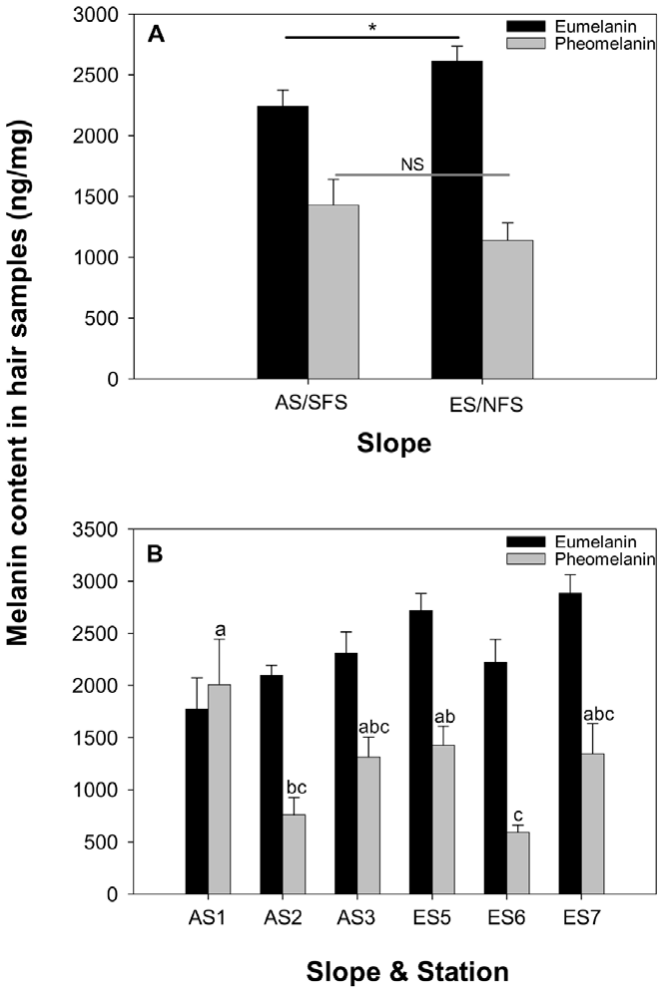
Contents of eumelanin and pheomelanin in hairs of spiny mice (*Acomys cahirinus*) from “Evolution Canyon” I, Israel. (A) in the opposite slopes, (B) across stations. Means with the same letter are not significantly different according to Waller-Duncan k-ratio t-test.

**Figure 5:**
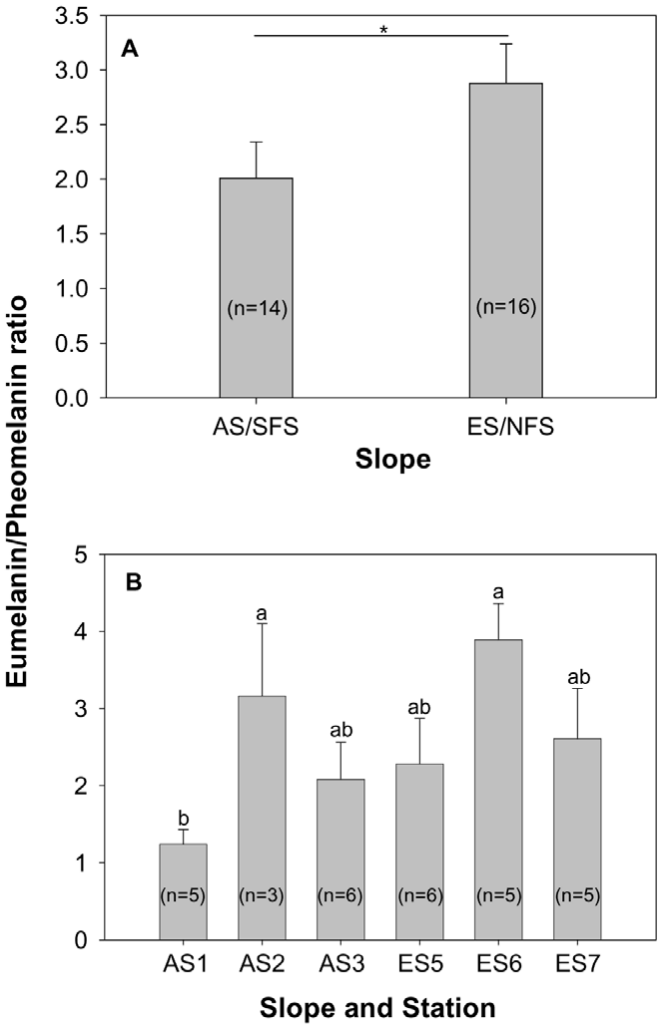
Eumelanin/Pheomelanin ratio of *A. cahirinus* from “Evolution Canyon” I, Israel. (A) in the opposite slopes, (B) across stations. Means with the same letter are not significantly different according to Waller-Duncan k-ratio t-test.

Hair melanin contents of *Acomys cahirinus* exhibit differences between the two (interslope) contrasting habitats. Mean eumelanin concentration in the hairs from ES/NFS individuals (2615±123 ng/mg; n = 16) was significantly (t-test, t_(28)_ = 2.063, P = 0.024; Figure 4A) higher than that from AS/SFS individuals (2243±132 ng/mg; n = 14). Although, mean pheomelanin contents from AS individuals' hairs (1429±213 ng/mg) were higher than that from ES individuals' hairs (1141±143 ng/mg), the difference was not significant (t_(28)_ = 1.145, P = 0.137; Figure 4A). The mean eumelanin/pheomelanin ratio is significantly (t_(28)_ = 1.759, P = 0.044; Figure 5A) higher in individuals from the ES (2.878±0.359) than those from the AS (2.010±0.331). Significant differences were obtained in pheomelanin contents (F_(5, 24)_ = 3.68, P = 0.0129; Figure 4B) and the eumelanin/pheomelanin ratio (F_(5, 24)_ = 2.70, P = 0.045, Figure 5B) of *A. cahirinus* captured from different stations of EC, while eumelanin (F_(5, 24)_ = 2.02, P = 0.111; Figure 4B) did not vary significantly between stations, but revealed the expected trend. The highest pheomelanin content was obtained for individuals from the AS1 station, while lowest pheomelanin content was obtained for individuals from the ES6 station (Figure 4B, Waller-Duncan k-ratio t-test). The highest eumelanin/pheomelanin ratio was obtained for individuals from the ES6 and AS2 station, while lowest eumelanin/pheomelanin ratio was obtained for individuals from the AS1 station (Figure 5B, Waller-Duncan k-ratio t-test). Comparisons of the melanin contents between male and female mice revealed that both eumelanin (males 2127±242 ng/mg, n = 6; females 2330±150 ng/mg, n = 8; t_(12)_ = 0.752, P = 0.467) and pheomelanin (males 1417±448 ng/mg, females 1437±198 ng/mg; t_(12)_ = 0.0448, P = 0.965) did not vary significantly on the AS. This was also true in the ES, wherein eumelanin (males 2811±159 ng/mg, n = 7; females 2462±170 ng/mg, n = 9; t_(14)_ = 1.462, P = 0.166) and pheomelanin (males 1233±187 ng/mg; females 1069±216 ng/mg; t_(14)_ = 0.552, P = 0.590) contents of males and females did not vary.

## Discussion

### Summary of results

The melanin pattern of spiny mice (*A. cahirinus*) approximates the background (habitat) color. Pheomelanin is insignificantly higher in individuals from the “African” slope to harmonize with the yellow to brownish background that is characterized by light terra rossa soil covered with a stretch of yellow-brownish grass across the slope (see Figure 2A); individuals on the “European” slope had significantly higher eumelanin content to mimic the dark (grayish) background caused by the shady, humus-laden, dark terra rossa soil habitat (Figure 2B). This evidence is further supported by the eumelanin and pheomelanin ratio, which is greater for the ES than the AS. Moreover, there are striking differences that exist across stations in hair-pheomelanin contents of *A. cahirinus*. This observation is noteworthy because rodent migration studies [38] found substantive upslope-downslope migration but none were found between slopes. Hair melanin variables did not differ significantly between males and females from both slopes. Thus, we reject the possible variation in hair melanin contents caused by sexual dimorphism or hair melanin pattern towards sexual selection in the species. Altogether, the hair melanin pattern of *A. cahirinus* supports the crypsis hypothesis and appears to respond to natural selection.

### Interslope hair-melanin divergence in EC I

How do spiny mice inhabiting the “African Slope (AS)” and “European Slope (ES)” vary in their melanin contents? The most likely explanation embraces a series of biochemical facts. Indeed, the pigmentation of hair in mammals mainly depends on the quantity, quality, and distribution of melanin. Natural melanin pigments consist of both insoluble brown to black eumelanin and alkali-soluble pheomelanin in varying ratios and are thus often considered “mixed melanin” [39]. Most melanogenesis leads to the production of copolymers (or mixtures) of eumelanin and pheomelanin. Taken together, spiny mice may modulate or (partially) switch the distribution of eumelanin and pheomelanin in the hairs. Such pigment switching is controlled by the interaction of two genes: the melanocortin-1 receptor (Mc1r), which encodes a seven-transmembrane receptor expressed in melanocytes, and its ligand, agouti, whose protein product is secreted from nearby dermal papilla cells and acts to inhibit Mc1r signaling. In the absence of agouti protein, Mc1r triggers intracellular cyclic adenosine monophosphate (cAMP) to activate eumelanin synthetic pathway. Conversely, the presence of agouti inhibits the activity of Mc1r, and thus the cAMP levels are reduced, resulting in lighter pigmentation. Therefore, it is the biochemical interaction between these two proteins that controls the switch between dark eumelanin and light pheomelanin production in melanocytes. Essentially the switching seems to be under genetic control [40]–[42].

One possible way by which the spiny mice modulate the amounts of eumelanin and pheomelanin, is adjusting the size/length of the agouti bands in hairs. Substantially, our results provide some clues that the concentration of pheomelanin fluctuated significantly between stations compared to that of eumelanin, which only led to significant variation in the eumelanin/pheomelanin ratio across stations. This indirectly signifies that the mice probably adjust the size of the agouti bands, or, in other words, they modulate the distribution and accumulation of pheomelanin in hairs to achieve background (habitat) matching. Presumably, the higher the pheomelanin contents derive from the greater the size/length of the agouti band [40]. In fact, the mice inhabiting the AS (Figure 3A and 3C) exhibit a markedly visible sub-apical agouti band, which is obvious from photographic images of the mice, living in a yellow-brownish background habitat. However, the same agouti band is present, but is not so visible in the images of mice inhabiting a dark grayish habitat on the ES (Figure 3B and 3D). Both eumelanin and pheomelanin derive from the common precursor dopaquinone that formed via the oxidation of L-tyrosine by the melanogenic enzyme tyrosinase. Intervention of cysteine leads to the production of pheomelanin. Therefore, the control of mixed melanogenesis might be based on tyrosinase activity and levels of L-tyrosine and L-cysteine [39].

*Acomys cahirinus* colonized Israel in the Upper Pleistocene, approximately 30–20,000 years ago [43]. Thus, the inter-slope color divergence and cryptic pigmentation in “Evolution Canyon” must have been evolving sometime after that period, which is relatively recent and fast. Although pigment switching seems to be under genetic control, information on other external/environmental stimuli that induces the switching has been studied the least. Conceivably, there could be other visual cues that animals might use to perceive the background color to exert such cryptic pigmentation. Despite the fact that mice have dichromatic vision, they might perceive the background based on its brightness. Yet, the means by which they perceive the light levels in their environment and how fast natural selection could transform these visual inputs into physiological execution of cryptic pigmentation is still unknown.

At the molecular level, melanocortin-1 receptor (product of *Mc1r* gene) and its antagonist, the agouti signaling protein (product of *Agouti* gene), are the two candidate genes responsible for most of the inter-specific as well as intra-specific differences in pigmentation [25], [26]. The production of eumelanin (brown or black pigment) and pheomelanin (yellow or red pigment) is controlled to a large part by the interaction of the two proteins [42], [44], [45]. Yet, mutations at one gene, the *melanocortin-1 receptor* (*Mc1r*), have been shown to be solely responsible in the majority of molecular underpinning of phenotypic variation. However, a recent study on melanism in *Peromyscus* showed independent mutations in agouti itself demonstrating that mutations in genes other than Mc1r can still produce changes in melanin [45].

### Interslope genetic (allozyme and DNA) divergence in Acomys at EC I

A previous study in our lab examined genetic allozyme and RAPD diversities to understand the ecological-genetic patterns in the spiny mouse *Acomys cahirinus* from the ecologically contrasting opposite slopes of “Evolution Canyon”, lower Nahal Oren, Israel [37]. Inter- and intraslope allozyme and RAPD divergence were found in *Acomys cahirinus*. Chord distance was used to measure the genetic distance according to Cavalli-Sforza and Edwards (1967) [46] (because Nei's genetic distances were too small to be used; D = 0.00–0.03). In C-S chord distance, populations are conceptualized as existing as points in a m-dimensional Euclidean space, which are specified by ‘m’ allele frequencies (i.e., ‘m’ = total number of alleles in both populations). The distance is the angle between these points. C-S chord distance assumes genetic drift only (no mutation). According to the C-S chord distance, AS1 and ES6 are closest ( = 0.051), whereas AS1 or AS2 and ES7 are distant ( = 0.103). There appears to be no gene flow between the populations of AS and ES. There is a large migration within each slope but almost no migration between the slopes (recapture rates in the alternate slope is zero) [38]. Considering all genetic results from that study, it appears that natural selection could be the primary cause for the differences in melanin content of the mice from opposing slopes of EC.

### Ecological and biochemical paradigms on coat coloration in mammals

The phenomenon of crypsis through their coat/pelage coloration in rodents is very well established for over a century [8], [47], [48]. Numerous studies endorse the perfection of coat/pelage coloration of rodents in response to the colors of the habitats in which they live [18]–[23]. Yet, all these studies measured the coat/pelage coloration using reflectance spectrometer. Our (pioneer) study on spiny mice is novel and unique in examining how melanin contents in the hairs evolve towards background matching. Studies on hair melanin contents in (small) mammals showed that eumelanin and pheomelanin values correlated well with the phenotypes [31], [49]. Several species of mammals, in addition to humans and mice, have been analyzed for their melanin contents. Black, yellow, and white areas of tortoise-shell guinea-pig hair were analyzed [50] and found to correspond to the eumelanin and pheomelanin contents. Aliev et al. (1990) [51] screened melanin contents in various colored wools obtained from several strains of sheep. The black phenotype resulted from eumelanin content, while tan and brown-to-red phenotypes resulted from mixtures of eumelanin and pheomelanin in varying ratios and levels. The results are also consistent for goats and llamas [52] and horses and other mammals [53]–[55]. However, these studies show hair melanin contents exclusively relate to phenotypes and not to crypsis or background matching.

Hair color is the most obvious and diverse characteristic among pigmentation phenotypes. The quality of habitats can influence animal color [56]–[58]. Coat coloration in nocturnal mammals is largely attributed to crypsis in a microgeographical scenario. Since the “Evolution Canyon” microsite exhibits both background color divergence and microclimatic divergence [33], it would be interesting to determine if the hair melanin pattern of *A. cahirinus* provides evidence for background matching and/or endorses Gloger's rule. Gloger's rule ascertains that darker individuals tend to reside in more humid regions and paler individuals in drier areas, possibly optimizing thermoregulation [59]. Although, we surmise that the hair melanin pattern in *A. cahirinus* may respond more towards background matching than microclimatic factors, we cannot rule out the possibility of both causes. Remarkably, regionally in Israel *A. cahirinus* is darker in the Galilee and lighter in the Negev desert and this is partly attributed to thermoregulatory processes [35], [60]. A recent study on coat color variation in house mice (*Mus musculus*) reveals that dark-colored coats of mice were observed in more humid and closed habitats with darker colored backgrounds, while pale coats were found in drier, more open habitats with lighter background color [23]. From our study and the findings of Lai et al. (2008) [23], we propose a combined hypothesis that ‘cryptic melanism’ and ‘thermal melanism’ in rodents may share a set of similar selection agents (e.g., open, dry habitats often represent light-colored background and closed, humid habitats often represent a dark-colored background).

### Conclusions and prospects

Based on our results with *A. cahirinus*, we propose the hypothesis that through natural selection rodents modulate or switch (so-called ‘pigment switching’) eumelanin and pheomelanin contents in their coat/pelage to achieve background matching in contrastingly colored habitats/backgrounds. Large-scale and critical studies that focus on cross-species comparison of hair melanin evolution towards background matching are needed to test our hypotheses. The role of *Mc1r* and *agouti* genes in effecting such pigment switching or modulation in spiny mice needs to be studied in detail. Furthermore, ecological transplant experiments and genetic crosses between mice of the opposite slopes of “Evolution Canyon” to assess the Mendelian genetics and levels of natural selection involved, are direct natural extensions of the current study.

## Materials and Methods

### Study site

“Evolution Canyon” I, located at lower Nahal Oren (32°43′N; 34°58′E), is a deeply erosive valley running from Mount Carmel westward into the Mediterranean Sea. The opposite slopes of the canyon have identical regional evolutionary history, geology, and soil type [32], [61], but they differ in topography (the “African” south facing slope dips 35°, the “European” north facing slope dips 25°). The slopes are separated by 100 m at the bottom and 400 m at the top. The climatic study in EC I [36] revealed that illuminance of the AS was significantly (2.3–8 times) higher than that of the ES; mean daily temperatures as well as daily temperature ranges were higher on the AS than on the ES, and, except under the high summer sun, relative humidity was 1–7% higher on the ES.

The “African” south-facing slope (AS) is covered by xeric, Mediterranean, savannoid formation of *Ceratonia siliqua*, *Pistacia lentiscus*, and savanna grasses (*Hyparrhenia hirta*, *Andropogon distachyos*, and *Pennisetum ciliare*). In contrast, the “European” north-facing slope (ES) is covered by an east Mediterranean maquis-forest (brushwood forest) of evergreen live oak of *Quercus calliprinos*, and *Pistacia palaestina* [62]. The remarkable interslope biotic divergence is illustrated by the fact that 62% of the higher plant species differ between the slopes and that the plant cover, making up the total cover of vegetation layers (measured by the Whittaker method), ranges from 35% (upper AS) to 150% (middle ES) [62]. The three stations on the “African” slope (1–3) exhibit African representative plants and animals taxa. By contrast, the three stations on the “European” slope (5–7) exhibit European representative plants and animals taxa [32].

### Animals and hair sampling

Individuals of spiny mice (*Acomys cahirinus*) were trapped from the opposing slopes (i.e., from both “African” and “European” slopes) of “Evolution Canyon” I, Nahal Oren, Carmel, Israel. A total of 30 mice were captured from the opposing slopes; 14 were caught from the AS including the upper, middle, and lower parts (stations AS1 (n = 5), AS2 (n = 3), and AS3 (n = 6), respectively), and 16 were caught from the ES (stations ES7 (n = 6), ES6 (n = 5), and ES5 (n = 5), respectively). Animals were housed in individual cages. They were kept under controlled conditions at 22–24°C and fed carrots and cucumbers. Animals used in this study were adults. The experiments were approved by the Ethics Committee of the University of Haifa. The mice were kept in the laboratory under 12∶12 LD (light/dark) cycle. Hair samples were carefully (i.e., to get the full length) excised with scissors from the rump region of animals over a 4×4 cm area, with uniform lengths (∼95% of total length). This procedure was consistent for all individuals to standardize sample collections for hair melanin analysis. Animals were treated humanely and care was taken while removing hairs to prevent injury or suffering.

### HPLC assays of eumelanin and pheomelanin in the hairs

For analysis of eumelanin and pheomelanin, hair samples were oxidized by permanganate to pyrrole-2,3,5-tricarboxylic acid (PTCA) and analyzed by HPLC with ultraviolet detection to determine eumelanin content [63]. To determine pheomelanin content, identical samples were hydrolyzed with hydriodic acid to 4-amino-3-hydroxyphenylalanine (4-AHP), and analyzed by HPLC with electrochemical detection [64]. Eumelanin assays were performed in duplicate while pheomelanin assays were only taken as single measurements. One ng of PTCA corresponds to 50 ng of eumelanin [65] while 1 ng of 4-AHP corresponds to 9 ng of pheomelanin [64].

### Statistics

The differences in hair melanin variables of *A. cahirinus* between AS/SFS and ES/NFS and between the sexes were compared using t-tests. Melanin contents are given as ng/mg. One-way analysis of variance (ANOVA), followed by the Waller-Duncan K-ratio t-test was used to compare the differences in hair-melanin contents of *A. cahirinus* across different stations in the opposing slopes of the EC. Data are shown as mean ± SE.
